# Molecular and physiological analysis of three *Pseudomonas aeruginosa* phages belonging to the “N4-like viruses”

**DOI:** 10.1016/j.virol.2010.06.011

**Published:** 2010-09-15

**Authors:** Pieter-Jan Ceyssens, Andrew Brabban, Larissa Rogge, Matthew Spooner Lewis, Derek Pickard, David Goulding, Gordon Dougan, Jean-Paul Noben, Andrew Kropinski, Elizabeth Kutter, Rob Lavigne

**Affiliations:** a Division of Gene Technology, Katholieke Universiteit Leuven, Kasteelpark Arenberg 21, Leuven, B-3001, Belgium; b Phage Biology Lab, The Evergreen State College, 2700 Evergreen Parkway NW, Olympia, WA 98505, USA; c The Wellcome Trust Sanger Institute, Genome Campus, Cambridge, CB10 1SA, UK; d Hasselt University, Biomedical Research Institute and Transnational University Limburg, School of Life Sciences, Diepenbeek, Belgium; e Laboratory for Foodborne Zoonoses, 110 Stone Road West, Guelph, Ontario N1G 3W4, Canada

**Keywords:** Bacteriophage, Genomics, Anaerobic infection, Pseudomonas aeruginosa, Phage N4, Mass spectrometry

## Abstract

We present a detailed analysis of the genome architecture, structural proteome and infection-related properties of three *Pseudomonas* phages, designated LUZ7, LIT1 and PEV2. These podoviruses encapsulate 72.5 to 74.9 kb genomes and lyse their host after 25 min aerobic infection. PEV2 can successfully infect under anaerobic conditions, but its latent period is tripled, the lysis proceeds far slower and the burst size decreases significantly. While the overall genome structure of these phages resembles the well-studied coliphage N4, these *Pseudomonas* phages encode a cluster of tail genes which displays significant similarity to a *Pseudomonasaeruginosa* (cryptic) prophage region. Using ESI-MS/MS, these tail proteins were shown to be part of the phage particle, as well as ten other proteins including a giant 370 kDa virion RNA polymerase. These phages are the first described representatives of a novel kind of obligatory lytic *P. aeruginosa-*infecting phages, belonging to the widespread “N4-like viruses” genus.

## Introduction

Bacteriophage N4, a lytic podovirus, was originally isolated from the sewers of Genoa, Italy, using *Escherichia coli* K-12 as host organism (Schito et al., 1967). N4 remained a genetic orphan for more than 40 years, being the only known phage that did not require the host RNAP for transcription of its early genes (Choi et al., 2008; Lavigne et al., 2008). The 70,153 bp genome (NC_008720) encodes 72 proteins and has direct terminal repeats varying in length between 390 and 440 bp (Ohmori et al., 1988). Remarkably, N4 uses three different RNA polymerases (RNAPs) during its life cycle, including a giant virion-encapsulated RNAP (vRNAP) of 3500 amino acids that is co-injected with the viral DNA upon phage infection. This vRNAP initiates RNA synthesis from three single-stranded hairpin promoters (Davydova et al., 2007a,b), which pulls the next 10–40 kb of the phage genome into the host cell. Injection of the remaining DNA requires transcription of the middle genes, carried out by a phage-encoded heterodimeric, T7-like RNAP which also requires an additional N4 protein, gp2 (Carter et al., 2003). One of the middle gene products, the N4 single-strand-DNA binding protein, activates the *E. coli* σ^70^-holoenzyme to carry out transcription from the late phage promoters (Beck et al., 2007).

Recently, phages DSS3ϕ2 and EE36ϕ1, infecting the marine roseobacter strains *Silicibacter pomeroyi* and *Sulfitobacter*, respectively, were reported to be related to N4 (Zhao et al., 2009). Also, we reported similarities between N4 and newly isolated *Pseudomonas aeruginosa* phages LIT1 and LUZ7 based on *de novo* analysis of structural phage proteins (Ceyssens et al., 2009). Here, we describe a detailed analysis of these two *P. aeruginosa* phages, and of the related phage PEV2. LIT1 and LUZ7 were isolated from Belgian hospital sewage samples using clinical *P. aeruginosa* strains US449 and Br257, respectively. In contrast, PEV2 was isolated by enrichment from the sewage treatment plant in Olympia, WA USA, and propagated on *P. aeruginosa* dog-ear strain PAV237.

### Phage characteristics

**Fig. 1:**
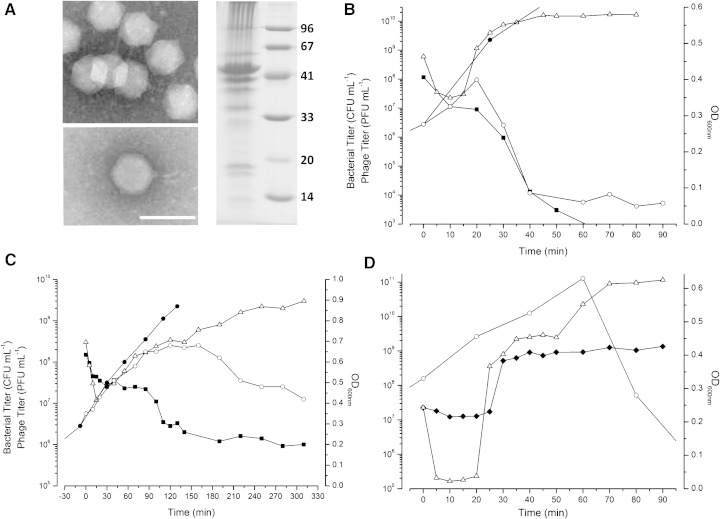
(A) Electron micrograph of LIT1 (top) and LUZ7 (bottom), next to the 1-DE protein profile of bacteriophage LIT1, prepared according to Moak and Molineux (2004) and loaded next to a protein marker (kDa). The scale bar represents 100 nm. High MOI (B and C) and low MOI (D) PEV1 infections of *P. aeruginosa* PAO1 growing in TSB (37 °C and 180 rpm) either aerobically (B and D) or anaerobically (C). Anaerobic cultures/infections were carried out in sealed serum vials under an N_2_ headspace in TSB supplemented with KNO_3_ (100 mM). All anaerobic manipulations were carried out using adaptations of the Hungate technique [Filiatrault et al., 2006; Raya et al., 2006). Symbols: OD_600 nm_ control (●), OD_600 nm_ infected, bacterial survivors cfu/ml (■), phage pfu/ml with (△) and without (♦) the addition of chloroform.

These phages are *Podoviridae*, having a 70 nm icosahedral capsid attached to a short tail at the characteristic portal vertex. A narrow 30 nm long tail structure is attached to the LIT1 capsid (Fig. 1A), but cannot be distinguished in LUZ7. They form clear plaques (1–2 mm diameter) surrounded by a small halo on lawns of their propagation hosts, can be amplified to high titers (> 10^11^ pfu/ml) in rich liquid media (LB/TSB) and are stable during long-term storage at 4 °C and upon purification with cesium chloride.

Studies of the phage infection process have been carried out at both high and low multiplicities of infection, anaerobically and aerobically. As seen in Fig. 1B and 1D, PEV2 has an eclipse period of about 20 min during aerobic infection at 37 °C in TSB. It lyses its host ∼ 25–30 min after infection, releasing ∼ 100–150 phage/cell. The OD_600 nm_ of the infected culture continues to increase almost as rapidly as for the uninfected control until just before lysis (Fig. 1B). Although seldom noted in the literature, such an increase in optical density is commonly observed for about one host-cell generation after infection with many lytic phages of both *E. coli* and *Pseudomonas.* These aerobic infection patterns are very different compared to the extended infection cycle reported for phage N4. Even though N4 encodes a lysozyme and holin, it does not actively lyse the host cell but rather allows the infected cells to continue growing for ∼ 3 h, asynchronously bursting and releasing up to 3000 phage particles per cell (Schito, 1974). Analogously, N4-like phages DSS3ϕ2 and EE36ϕ1 have latent periods of 3 and 2 h and yield 350 and 1500 progeny, respectively, at 28 °C in a yeast-tryptone broth medium (Zhao et al., 2009).

Virtually all published characterizations of phage infection processes have been carried out in rapidly growing cells under aerobic conditions, even when the hosts are actually facultative aerobes and their site of pathogenesis is anaerobic or microaerophilic. A few anaerobic studies have been reported for T4-like and T5-like coliphages, where significant differences in infection parameters are often observed (Kutter et al., 1994; Raya et al., 2006). *Pseudomonas* is capable of growing very well anaerobically in mucus (∼ 10^8^ cfu/ml after 72 h) when supplied with the external electron acceptors nitrate or nitrite, both of which are present at relevant concentrations in the sputa of cystic fibrosis (CF) patients (NO_3_^-^ ∼ 0.4 mM and NO_2_^-^ ∼ 0.1 mM). Moreover, *P. aeruginosa* growing in anaerobic or microaerobic biofilms in the lungs is responsible for ∼ 95% of CF patient deaths (Jones et al., 2000; Hassett et al., 2002). It is therefore particularly important to look anaerobically at phages being considered for many therapeutic applications. PEV2 is indeed able to infect PAO1 growing anaerobically, although the replication cycle proceeds more slowly (Fig. 1C). The observed eclipse period under anaerobic conditions increased to 60 min, and lysis was more gradual. The reason for the slow lysis is not clear, as some lysozyme is produced within 12 min of infection as indicated by cell susceptibility to lysis by chloroform (data not shown). A fraction of the infected cells lysed around 100 min after infection, as reflected in the killing of most remaining bacterial survivors at that time. However, the OD did not drop significantly until ∼ 200 min after infection and only 20 phages per cell were eventually produced. Similar patterns of anaerobic and aerobic infection were seen in all tested sensitive CF and dog-ear strains, and PEV2 is currently being tested in the treatment of dog-ear infections as well as in a typing set for characterizing clinical strains from CF patients.

### General genome characteristics

**Fig. 2:**
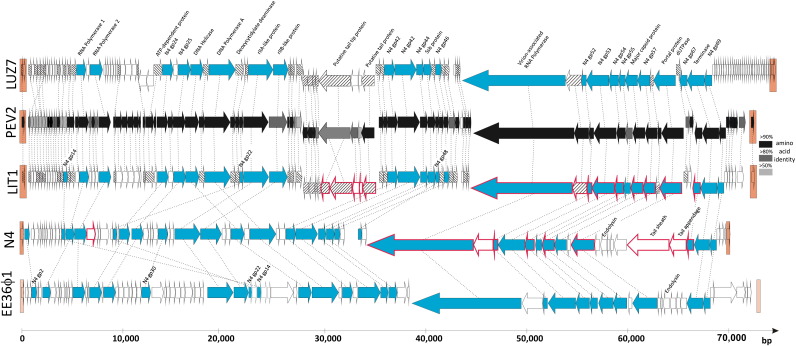
In silico analysis and comparison of five N4-like phage genomes. The arrows represent the predicted ORFs, and point at the direction of transcription. The direct repeats delineating the genomes (which were not reported for EE36ϕ1) are indicated as orange boxes. Genes which are shared in all N4-like phages are colored blue, genes unique to *Pseudomonas*-infecting N4-likes are hatched and genes sharing amino acid similarity are connected with broken lines. The amino acid identity between phages LIT1 and PEV2 is indicated in different shades of grey. Finally, genes encoding the structural proteins which were experimentally identified are outlined in red, and compared to the genomic location of structural N4 genes (Choi et al., 2008). As roseophage DSS3ϕ2 is very similar to EE36ϕ1 (Zhao et al., 2009), it is not included in this schematic drawing. The genomes of LUZ7 and LIT1 were deposited in GenBank under accession numbers NC_013691 and NC_013692NC_013691NC_013692, respectively.

Using traditional Sanger methods for sequencing of shotgun libraries and primer walking, the entire genome sequences of the three *Pseudomonas* phages were determined with at least 10 times sequence coverage, and annotated as described elsewhere (Ceyssens et al., 2008). Phages LUZ7, LIT1 and PEV2 encapsulate linear dsDNA genomes of 74,901 bp, 72,543 bp and 72,697 bp, respectively, with G + C contents varying between 53.4 and 54.9%. All three genomes are delineated by relatively long (632 to 665 bp) direct terminal repeats (Suppl. Figure S1 for LUZ7). Despite being isolated by different researchers on different continents, phages PEV2 and LIT1 exhibit 94% identity in nucleotide sequence, share similarity in each encoded gene except for the tail fiber, and can therefore be considered as different isolates of the same phage species. In contrast, LUZ7 and LIT1/PEV2 have an overall nucleotide similarity of 58% as determined by the Stretcher algorithm (Myers and Miller, 1988), and display protein similarity in only 60 of their gene products (Fig. 2).

The genomes of LUZ7 and LIT1/PEV2 encode 117 and 92 proteins, respectively (Suppl. Table 1). The difference in gene number between these two groups of phages is largely due to an extra cluster of 29 small genes, located directly upstream from the right terminal repeat of the LUZ7 genome (Fig. 2). These genes lack any similarity to current database entries and any recognizable structural features, so their functions remain unknown. Phages LUZ7 and LIT1/PEV2 clearly share their overall genomic architecture as well as 25 core gene products with coliphage N4 and with roseophages EE36ϕ1 and DSS3ϕ2, strongly suggesting a common origin for these phages. While gene order among most structural genes is strictly conserved, major rearrangements have occurred among DNA replication, tail formation and host lysis genes (Fig. 2; e.g., N4-like ORFs 14 and 22). In contrast to N4 which encodes three tRNA genes, none are predicted in LUZ7 or LIT1/PEV2.

### N4-like transcriptional strategy

Based on *in silico* analysis, mass spectrometry data and their analogy with N4, we predict that three different RNA polymerases successively carry out transcription of early, middle and late genome regions. As seen by mass spectrometry, the LUZ7 and LIT1/PEV2 particles contain a giant vRNAP (3,398 amino acids) (Table 1) which is devoid of cysteine residues; this could be an important requirement for the enzyme to pass through the tail into the cell and/or through the host periplasm, which contains proteins that catalyze disulfide bond formation (Ritz and Beckwith, 2001). Inspection of the early genome region revealed three potential hairpin promoters, preceding genes 1, 12 and 13, each composed of a 4–5 nt hairpin and a 3 nt loop (Suppl. Table S2). These are structurally similar and analogously located compared to the 3 early N4 promoters reported by Glucksmann et al. (1992), which make two essential sequence-specific contacts at the stem (−8G) and at the central purine of the hairpin loop (−11G) (Davydova et al., 2007a,b). The subtle differences of sequence and hairpin-stem length among these early promoters would be expected to affect the affinity of vRNAP binding, as described in N4 (Gleghorn et al., 2008).

LUZ7, LIT1 and PEV2 each encode a heterodimeric RNA polymerase which contains all essential catalytic residues of N4's T7-like RNAP (Suppl. Figure S2) for middle transcription, as well as a homologue of N4's ssDNA-binding protein, which redirects the host RNAP to transcription from late phage promoters (Miller et al., 1997). At least ten rho-independent terminators could be identified throughout the phage genomes (Suppl. Table S2).

### Particle composition and host lysis

To analyze the protein content of the LIT1/PEV2 particle, we separated virion proteins of doubly CsCl-purified phages using 1-D SDS-PAGE (Fig. 1A). Entire gel lanes were cut into slices and subjected to an overnight trypsin digestion. Peptides were separated by liquid chromatography with a linear 5–60% (vol/vol) acetonitrile gradient and subsequently identified by ESI-MS/MS (LCQ Classic, Thermonfinnigan) in an *m/z* range of 300–1500. All MS data were analyzed using Sequest (Thermofinnigan) considering a minimal cross correlation value of 1.8, 2.5 and 3.5 for single, double or triple charged peptide ions, respectively (Lavigne et al., 2006). In this way, 14 proteins were shown to be part of the mature phage virion (Table 1, Fig. 2). In comparison, the mature virion of N4 is reportedly composed of ten gene products, including the major capsid protein (*gp56*), the vRNAP (*gp50*), the tail sheath protein (*gp65*), the appendages (*gp66*) and the portal protein (*gp59*) (Fig. 2). The N4 capsid is decorated with 175 copies of gp17 (Choi et al., 2008).

The *Pseudomonas* phages lack N4's head decoration protein and show very significant structural differences from N4 in the tail. In phages LIT1/PEV2 and LUZ7, the entire cluster of N4 tail genes is apparently replaced by a cluster of genes embedded (in reverse orientation) in the replication module (ORFs 52–59 in LUZ7, ORFs 48–56 in LIT1; Fig. 2). These proteins display strong similarity to *P. aeruginosa* prophage proteins and various tail proteins from other *Podoviridae* (Suppl. Table S1), and four of these proteins (*gp52–56*) were identified as part of the phage virion of LIT1 (Table 1). As such, an ancestor of the N4-related *Pseudomonas* phages seems to have acquired its host recognition proteins from a prophage region in the *Pseudomonas* host genome (nt 698932–703058 in PAO1). Interestingly, these proteins are part of a bacterial gene cluster which is significantly upregulated upon stress conditions and phage infection (Lecoutere E., personal communication*).* Despite the common origin of the tail genes, a few notable differences are present, like the fusion of ORF52 and ORF53 of phage LIT1 into a single, larger gene (ORF56) in phage LUZ7 (Suppl. Table S1). This may account for the observed differences in tail morphology between these phages. In contrast to their homologues in N4, gp74 and gp78 are also identified as structural proteins in the N4-like *Pseudomonas* phages.

Although rapid host lysis is observed at least aerobically (Fig. 1B), no putative muralytic enzyme could be predicted *in silico*, suggesting a novel type of endolysin or lysis mechanism. The genome region encoding the N4 acetylmuramidase (Stojković and Rothman-Denes, 2007) and the EE36ϕ1 endolysin is clearly deleted in both LUZ7 and LIT1/PEV2 (Fig. 2). Future research will target candidate lysin genes near possible holins, i.e., small proteins carrying membrane-spanning domains (e.g., LUZ7 ORFs 48 and 49).

### Concluding remarks

Although phage N4 was a genetic orphan for over 40 years, it is now apparent that “N4-like viruses” are widespread in nature. A number of phages of this genus have been isolated in Olympia against CF and dog-ear *P. aeruginosa* strains, and new N4-related phages infecting *E. coli, Roseobacter, Vibrio, Klebsiella* and *Salmonella* species are currently under investigation in laboratories worldwide (18th Evergreen International Phage Biology Meeting, August 2009). This group of obligatorily lytic phages has a conserved genome architecture, with a distinguishing three-step transcription mechanism initiated by a giant virion-encapsulated RNAP. The observed *Pseudomonas*-specific modifications of LUZ7 and LIT1/PEV2 have triggered interest in a specific prophage region of *P. aeruginosa*, which is currently being investigated using micro-array based methods.

## Figures and Tables

**Table 1:** Mass spectrometric identification of structural proteins of phage LIT1.

Gp	Mass (kDa)	No. peptides	Coverage (%)	Closest homologue	*E*-value	Proposed role in N4^a^	No. N4 copies^b^
52	29.8	2	16.4	PA0646 [*P. aeruginosa* PAO1]	6e–16	Absent	
53	79.5	13	41.9	Tail fiber [*Yersinia* phage Berlin]	5e–10	Absent	
54	25.0	3	30.4	PA0642 [*P. aeruginosa* PAO1]	0.1	Absent	
55	11.7	2	47.7	none		Absent	
56	46.4	7	31.0	PA0641 [*P. aeruginosa* PAO1]	3e–5	Absent	
71	370.1	76	46.7	gp50 [N4]	2e–13	Virion RNAP	1 or 2
72	56.7	14	61.1	none		Structural protein	18 ± 1
73	16.9	2	24.5	gp52 [N4]	0.18	Structural protein	33 ± 5
74	82.1	5	13.9	gp53 [N4]	1e–10	N.I.	
75	35.4	11	60.7	gp54 [N4]	2e–7	Structural protein	43 ± 9
77	44.2	16	82.7	gp56 [N4]	2e–121	Major capsid	535
78	44.9	1	3.0	gp57[N4]	3e–34	N.I.	
80	80.7	21	69.1	gp59[N4]	0	Portal protein	12
83	28.0	4	42.6	gp67[N4]	3e–9	Structural protein	13 ± 5

a N.I. Homologues are present in N4, but were not identified as part of the N4 particle (Choi et al., 2008).

b As determined/reported previously (Choi et al., 2008).

## Appendix A Supplementary data

Supplementary Figure S1 Determination of the exact length of the direct terminal repeats of phage LUZ7 by direct sequencing with outward-directed primers. Primers (horizontal arrows) annealing outside the DTR (white boxes) lead to a stop of the sequencing reaction at the ends of the genome (blue boxes), while primers annealing within these repeats lead to a 50% reduction in signal intensity. The defined ends of the LUZ7 are indicated with vertical arrows.

Supplementary Figure S2 Amino acid sequence alignment of four Motifs (T/DxxGR, A, B and C) between RNA Polymerase genes encoded by N4-like viruses, compared to other phages of the *Autographivirinae*. Residues highlighted in red are identical in all these phages, residues that highlighted in yellow are > 50% identical. Motifs A and C contain the aspartate residues for chelating the catalytically essential Mg^2+^ ions, while Motif B (Rx_3_Kx_7_YG) binds the triphosphate moiety of the incoming nucleotide. The only exception is the Y678F substitution, which possibly aids in discrimination against dNTP incorporation (Kazmierczak et al., 2002).

Supplementary Table S1 *In silico* analysis of the sequenced phage genomes. Only the ORFs displaying similarity to database entries or encoding structural features are shown.
