# Supplementary material for: Molecular and physiological analysis of three Pseudomonas aeruginosa phages belonging to the “N4-like viruses”
Source: Virology. 2010 Sep 15;405(1):26–30. doi: 10.1016/j.virol.2010.06.011 (PMC3715699; doi:10.1016/j.virol.2010.06.011)
Supplement: Supplementary Figure S1 — Determination of the exact length of the direct terminal repeats of phage LUZ7 by direct sequencing with outward-directed primers. Primers (horizontal arrows) annealing outside the DTR (white boxes) lead to a stop of the sequencing reaction at the ends of the genome (blue boxes), while primers annealing within these repeats lead to a 50% reduction in signal intensity. The defined ends of the LUZ7 are indicated with vertical arrows. [file mmc1.pdf]

SUPPLEMENTARY FIGURE S1

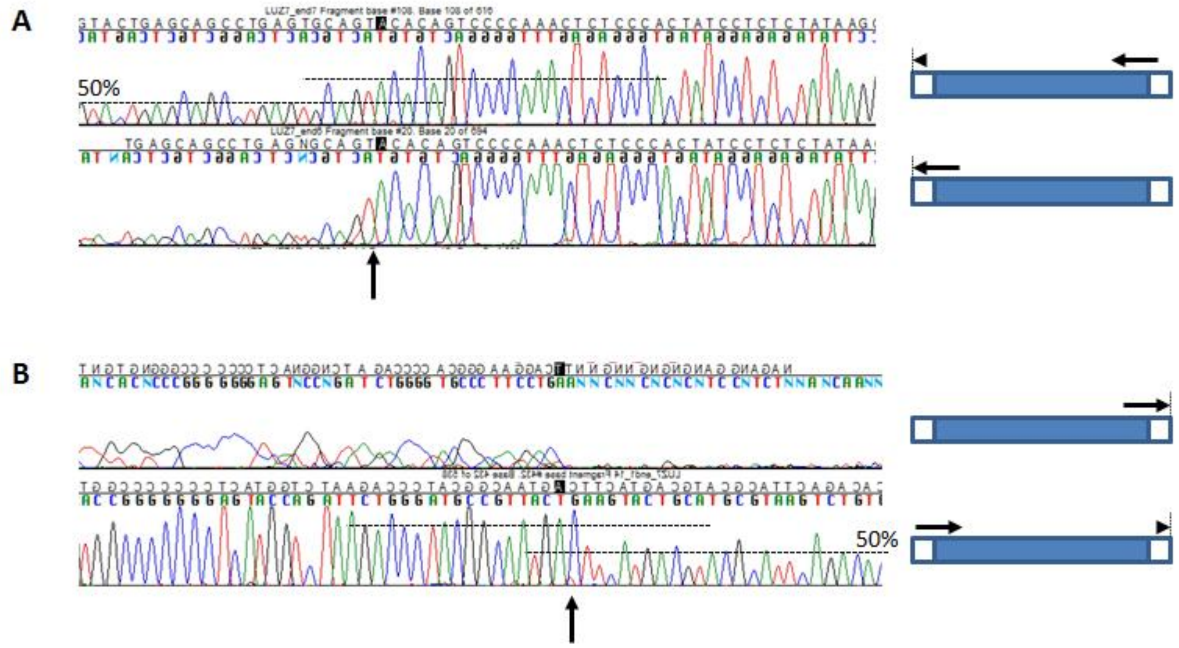

Supplementary Figure S1. Determination of the exact length of the direct terminal repeats of phage LUZ7 by direct sequencing with outward-directed primers. Primers (horizontal arrows) annealing outside the DTR (white boxes) lead to a stop of the sequencing reaction at the ends of the genome (blue boxes), while primers annealing within these repeats lead to a 50% reduction in signal intensity. The defined ends of the LUZ7 are indicated with vertical arrows.

SUPPLEMENTARY FIGURE S2

Motif A

```
T7            LPLAFDGSCSG-IQHFSAMLRDEVGGRAV-NLLP
gh-1          LPIAFDGSCSG-IQHFSAMLRDERGGRAV-NLLQ
phiA1122      LPLAFDGSCSG-IQHFSAMLLDEVGGLAV-NLLP
K11           LPLAFDGSCSG-IQHFSAMLRDSIGGRAV-NLLP
K1F           LPIAFDGSCSG-IQHYSAMLRDHIGGHAV-NLTP
K1-5          LPVHQDGSCSG-IQHYSAMLRDAVGAKAV-NLKP
Era103        LPVHQDGSCSG-IQHYSAMLRDKTGAKAV-NLMP
VP4           LPLAFDGTCSG-LQHFSAMLRDEIGGAAV-NLLP
phiKMV        FIVHMDATCSG-LQHYSAILRDEIGGAYV-NLLP
XP10          VSADMDGTCNG-LQHFSAMLRDEIGAAAT-NLVP
SP6           LPVHQDGSCSG-IQHYSAMLRDEVGAKAV-NLKP
P60           LPIGIDATCSG-LQHLSSMTRDAVAAKQV-NVIR
Syn5          LPIGIDATCSG-LQHLAAMTRCGRTAALV-NVTP
P-SSP7        LPVATDATCSG-LQILAGLARDKSTASKV-NVIP
LIT1 RNAPII   HLVGLDACCSG-MQIMSALGGCESGAQAT-NLIN
LUZ7 RNAPII   HLVGLDACCSG-MQIMSALGGCIDGARAT-NLVN
PEV2 RNAPII   HLVGLDACCSG-MQIMSALGGCESGAQAT-NLIN
N4            LYVEADGVTNGPINAMMLMTGGLFTPDWIRNIAK
DSS3ϕ2        LSFELDGLTNGAANMMINFGHGLMTPEDWGNFKR
EE36ϕ1        LSFELDGLTNGAANMMINFGHGLMTPEDWGNFKR
LIT1          LLGEVDGVTNG-PMLSLLMSGAKGFDTLNQGGFF
LUZ7          LLGEVDGVTNG-PMLSLLMSGAKGFETLNQGGFF
PEV2          LLGEVDGVTNG-PMLSLLMSGAKGFDTLNQGGFF
```

Motif B

```
T7            KALAGQWLAYGVTRSVTKRSVMTLAYGSKEFGFRQQVLEDTIQPAIDSGKG
gh-1          KTLAMAWLTYGMSRKVTKRSVMTLAYGSKAYGFADQVREDIVKKAIDNGDG
phiA1122      KALAGQWLAYGVTRSVTKRSVMTLAYGSKEFGFRQQVLEDTIQPAIDSGKG
K11           SVLAAQWLQYGVTRKVTKRSVMTLAYGSKESLVRQQVLEDTIQPAIDNGEG
K1F           RELARQWLTYGMSRKVTKRSVMTLAYGSKEYGFADQVYEDIVMPAIDSGSG
K1-5          RSMASAWDMIGITRGLTKKPVMTLPYGSTRLTCRESVIDYIVDLEEKEAQR
Era103        KAMAESWDAIGITRSLTKKPVMTLPYGSTRITCRESIDDYLVSLEEFELRK
VP4           RTLAAQWLEYGVTRSVTKRSVMTLAYGSKEYGFADQVFEDTVMPAIDNGKG
phiKMV        ------WDKAGLSRSLTKKPCMTLVYGTTFKGVVDHCLDYLDESGVEIPEG
XP10          -GLVEKWLQHGINRSITKRSTMTLPYGSTRFSSAGFVRDDYLL----KGKA
SP6           -------IHPYMNRKITKRTVMTVPYGVSRDSARAYIREALHDAGFDLSTK
P60           RAMASAWDSIGITRSLTKKPVMTLPYGSTRLTCRESVIDYIVDLEEKEAQK
Syn5          ----------WITRKVTKRPVMCTPYGVTMSSARGYIRDQLVK----DGHK
P-SSP7        -----ERLRPYWDRKKTKRCVMTIPYNAKPFSNRQYIRDAFEDIDIEVEND
LIT1 RNAPII   ASTHLGKSVGEIKRSDIKTATMTHFYGSKAEPKNLFGEDTPELEAFYQGVE
LUZ7 RNAPII   ASTAMGASVGDIKRSDIKKATMTHLYGSKAEPKKLFGEETPELEAFYEGIQ
PEV2 RNAPII   ASTHLGKSVGEIKRSDIKTATMTHFYGSKAEPKNLFGEDTPELEAFYQGVE
N4            DINLGENGALELKRGIAKNPLTITIYGSGARGIAGKLVSSVTDAIYERMSD
DSS3ϕ2        FEMDTTTGDFWMTRNTAKNPMTKVNYGSGVQGVAIGVADDMLLEFYEKLQD
EE36ϕ1        FEMLPN-GDFKMTRNTAKNPMTKVNYGSGVQGVAIGVADDMLLEFYEKLQT
LIT1 vRNAP    VLTNEEGGVSSKGRNVIKKPLTAMMFGSNTKTAVSGMADAFIETIYSKMED
LUZ7 vRNAP    ELSTDDGNVSSKGRKVIKQPLTAMMFGSNTKTAVDGMADGFIETIYSKIED
PEV2 vRNAP    VLTNEEGGVSSKGRNVIKKPLTAMMFGSNTKTAVSGMADAFIETIYSKMED
```

T/DxxGR

```
T7            LEQANKFANHKAIWFPYNMDWRGRVYAVSMFN-PQGNDMTKGLLTLAKG
gh-1          LEQANKFAEYDAIYFPYNLDWRGRVYAIPAFN-PQSNDMTKGILQAAKG
phiA1122      LEQANKFANHKAIWFPYNMDWRGRVYAVSMFN-PQGNDMTKGLLTLAKG
K11           VAQANKFANHKAIWFPYNMDWRGRVYAVSMFN-PQGNDMTKGSLTLAKG
K1F           VNQANKFSQFKAIWFPYNMDWRGRVYAVPMFN-PQGNDMQKGLLTLAVG
K1-5          VGQARKYSQFDAIYFVYALDSRSRVYAQSSTLSPQSNDLGKALLRFTEG
Era103        LNQASDLAKFESIYFVYAMDSRGRVYVQSSGVSPQSDDLGKSLLRSTKG
VP4           LSQANKFSKYNEIYFPYNMDSRGRVYAIPMFN-PQGNDMVKGLLTFAKK
phiKMV        LYRVVREFFGKAVYFPMHVDSRGRMYYWGTPN-PQGSDIAKACLRFHDK
XP10          RTVANKFLAYPAIYFVYFCDFRGRKYAMGSGINPQGSDLQKCLLEFAKG
SP6           VGQARKYSAFESIYFVYAMDSRSRVYVQSSTLSPQSNDLGKALLRFTEG
LUZ7 RNAPII   EMYKLMVQQGNKFYLLHKVDKRGRLYAQGYHISTQGASYKKAMIDLYNK
LIT1 RNAPII   EMYKLMVQQGNRFYLLHKYDKRGRVYAQGYHISTQGSPYKKAMLDLANK
PEV2 RNAPII   EMYKLMVQQGNRFYLLHKYDKRGRVYAQGYHISTQGSPYKKAMLDLANK
P60           MFVANQYDDEV-FWIPWSFDYRGRVYPQNTQLNPQGTDFDKSLLLFAEE
Syn5          MYVARKYADEASFWMPASFDYRGRVYFLNTALNPQGTDFDKALLYFAEE
P-SSP7        MNCVREFKDKE-YYIPWSFDYRGRAYPIPSFLTPQDTDFGKSLIRFAEE
N4            VRAQSEDISTVPIHYAYNMTRVGRMQMLGKYN-PQSAKLVREAILPTKA
DSS3ϕ2        VNGMMEQQASE-VFFPVGVTKVGR-HQMQGPN-PQNNKLMRYVVAPQRS
EE36ϕ1        INGMMEQQASE-VFFPVGVTKVGR-HQMQGPN-PQNNKLMRYVVAPQRS
LIT1 vRNAP    IQEVSDLGVEQPLYFDRSVWKPQRVGLTANMINPQTSKVHRHMLAMEGW
LUZ7 VRNAP    INEVSDLGLEQPLYFDRSVWKPQRVGLTSNLINPQTSKVHRHMLKMEGW
PEV2 vRNAP    IQEVSDLGVEQPLYFDRSVWKPQRVGLTANMINPQTSKVHRHMLAMEGW
```

Motif C

```
T7            KYGIESFALIHDSFGTIPAD
gh-1          RYGIEFFALIHDSFGTIPAH
phiA1122      KYGIESFALIHDSFGTIPAD
K11           VYGIDSFALIHDSSGTIPAD
K1F           KYGVMSFAVIHDSFGTIPAD
K1-5          K-GITSIAVIHDSFGTHAGR
Era103        A-GLEFVAVIHDSFGTLACD
VP4           VYGIHSFAMIHDSFGCHAGF
phiKMV        E--DIPIQAIHDSMGTYASD
XP10          V-GIKDFAMIHDSYAVHCCH
SP6           K-GVTSIAVIHDSFGTHADN
P60           P-----FTVIHDCILGRSCD
Syn5          P-----FTVIHDCVLGRSCD
P-SSP7        P-----IALIHDSVLCRATD
LIT1 vRNAP    ALMGEDVLNIHDAHGVGVLG
LUZ7 vRNAP    ALDGASVLNIHDAHGTGLTD
PEV2 vRNAP    ALMGEDVLNIHDAHGVGVLG
LIT1 RNAPII   VHEPFELVTVHDEFKCHPNF
LUZ7 RNAPII   AHKPFPLVTVHDEFKCHANH
PEV2 RNAPII   VHEPFELVTVHDEFKCHPNF
N4            KGAPKNTLKIFDGMNIGLND
DSS3ϕ2        DNAPNDVLPVFDGIDVPVSK
EE36ϕ1        DNAPNDVLPVFDGIDVPVSK
```

Supplementary Figure S2. Amino acid sequence alignment of four Motifs (T/DxxGR, A, B and C) between RNA Polymerase genes encoded by N4-like viruses, compared to other phages of the *Autographivirinae*. Residues highlighted in red are identical in all these phages, residues that highlighted in yellow are > 50% identical. Motifs A and C contain the aspartate residues for chelating the catalytically essential Mg^{2+} ions, while Motif B (Rx_3Kx_7YG) binds the triphosphate moiety of the incoming nucleotide. The only exception is the Y678F substitution, which possibly aids in discrimination against dNTP incorporation (Kazmierczak *et al.*, 2002).

SUPPLEMENTARY TABLE S1. *In silico* analysis of the sequenced phage genomes. Only the ORFs displaying similarity to database entries or encoding structural features are shown.

ORF LUZ7	ORF LIT1	Mut. ident.[a]	Mw (kDa)	Strand	Closest homologue	E-value[b]	Comments
**1**	**1**	33	9.9	+	No similarity		Contains signal peptide (P=0.98)
**2**	**-**	-	12.3	+	No similarity		Two transmembrane domains
**3**	**5**	35	10.7	+	No similarity		
**5**	**6**	25	12.4	+	No similarity		
**6**	**6**	26	12.3	+	No similarity		
**7**	**7**	40	11.8	+	No similarity		
**8**	**2**	27	9.1	+	No similarity		
**-**	**8**	-	7.3	+	No similarity		Two transmembrane domains
**-**	**9**	-	7.9	+	No similarity		Two transmembrane domains; signal peptide (P=0.98)
**-**	**10**	-	8.5	+	No similarity		Two transmembrane domains
**-**	**11**	-	8.0	+	No similarity		Two transmembrane domains
**10**	**-**	-	3.6	+	No similarity		One transmembrane domains; signal peptide (P=0.9)
**12**	**-**	-	8.4	+	No similarity		C-terminal transmembrane domains
**13**	**-**	-	7.1	+	No similarity		Two transmembrane domains
**14**	**13**	37	11.1	+	No similarity		
**16**	**14**	19	8.7	+	No similarity		
**-**	**15**	-	14.6		gp14 [N4]	2e-23	
**18**	**16**	49	9.5	+	No similarity		
**19**	**17**	50	15.7	+	No similarity		
**20**	**19**	72	35.9	+	RNAP1 [N4]	6e-23	C-terminus: T3/T7-like RNA polymerase [PHA00452, 4e-22]
**22**	**23**	69	46.9	+	RNAP2 [N4]	2e-45	N-terminus: DNA-dependent RNA polymerase [pfam00940, 2e-14]
**29**	**24**	43	7.7	+	No similarity		Two transmembrane domains; signal peptide (P=0.99)
**30**	**-**	-	18.3	+	No similarity		TraB pilus assembly protein [pfam06447, 2e-3]
**33**	**30**	69	6.7	+	No similarity		
**35**	**-**	-	23.5	-	gp36 [phig1e]	7e-08	
**36**	**31**	46	30.7	+	Clp Protease [*P. uenosis* 60-3]	2e-15	ATPase family associated with various cellular activities [pfam07724, 3e-8]
**-**	**32**	-	9.1	+	hypothetical [*P. fluorescens*]	3e-5	
**37**	**33**	48	38.8	+	gp24 [N4]	1e-61	ATPase family associated with various cellular activities [pfam07728, 3e-5]
**39**	**35**	41	44.6	+	gp25 [N4]	3e-34	Predicted metallopeptidase [pfam09967, 3e-9]
**40**	**36**	51	46.6	+	Helicase [N4]	1e-31	
**41**	**37**	33	20.3	+	No similarity		
**42**	**38**	65	98.4	+	DNA Polym. [N4]	0.0	DNA polymerase I - 3'-5' exonuclease and polymerase domains [COG0749, 9e-38]
**43**	**39**	55	21.4	+	hypothetical [*T. kodakarensis*]	2e-15	Deoxycytidylate deaminase [cd01286, 1e-19]
**45**	**40**	38	7.2	+	No similarity		
**-**	**41**	-	13.2	+	gp22 [N4]	1e-31	
**46**	**42**	30	94.6	+	rIIA [N4]	2e-51	Only N-terminal part conserved
**47**	**43**	44	55.2	+	rIIB [N4]	8e-57	Central part not conserved
**48**	**44**	29	8.8	+	No similarity		Two transmembrane domains
**49**	**45**	62	11.9	+	No similarity		Three transmembrane domains
**51**	**47**	14	22.3	+	No similarity		
**52**	**48**	68	17.8	-	tail protein [E1]	1e-3	Protein of unknown function [pfam10721, 6e-8]
**53**	**49**	60	19.2	-	hypothetical [*D. acidovorans*]	1e-18	
**54**	**50**	43	9.2	-	PA0648 [*P.aeruginosa*]	2e-25	
**55**	**51**	27	10.7	-	PA0647 [*P. aeruginosa*]	1e-33	
**56**	**52**	**(C-term)**	115.8	-	PA0646 [*P. aeruginosa*]	6e-16	N-terminus similar to various tail fiber proteins
	**53**	**(N-term)**			Tail fiber[Berlin]	5e-10	C-terminus: SGNH_hydrolase [cd00229, 2e-5]
**-**	**54**	-	25.0	-	PA0642 [*P. aeruginosa*]	0.1	
**59**	**56**	63	46.3	-	tail tip fiber	7e-12	Related to C-terminal part of many phage tail fibers

(Table S1. Continued)

					[phage 86]		Phage-related tail component [COG4733, 3e-4]
**60**	**57**	70	15.5	+	No similarity		
**61**	**58**	47	14.9	+	No similarity		
**62**	**59**	63	36.9	+	gp42 [N4]	3e-78	
**63**	**60**	65	81.1	+	gp43 [N4]	0.0	
**64**	**61**	63	28.0	+	gp44 [N4]	8e-61	
**65**	**62**	42	26.7	+	gp45 [N4]	6e-24	Single-stranded DNA-binding protein
**66**	**63**	76	13.7	+	ORF144 [P. damselae]	1e-12	
**-**	**64**	-	17.1	+	gp48 [N4]	9e-12	Hypothetical protein [PHA01971, 3e-16]
**67**	**65**	61	20.0	+	gp46 [N4]	8e-38	
**68**	**66**	28	13.0	+	No similarity		
**69**	**67**	38	14.2	+	No similarity		Signal Peptide (P=0.98)
**71**	**69**	27	12.8	+	No similarity		
**72**	**70**	38	7.1	+	No similarity		Two transmembrane domains
**73**	**71**	65	370.1	-	vRNAP [N4]	1e-87	Only C-terminal and central part conserved N-terminus: TolA-like domain [PRK09510, 1e-3]
**74**	**72**	49	56.7	-	No similarity		
**75**	**73**	70	16.9	-	gp52 [N4]	0.089	One transmembrane domains
**76**	**74**	51	82.1	-	gp53 [N4]	1e-10	Three transmembrane domains
**77**	**75**	58	35.4	-	gp54 [N4]	1e-16	
**78**	**76**	49	24.1	-	gp55[N4]	2e-24	
**79**	**77**	77	44.2	-	gp56 [N4]	2e-121	Major coat protein
**80**	**78**	46	44.9	-	gp57[N4]	1e-36	
**81**	**79**	24	12.8	-	No similarity		
**82**	**80**	63	80.7	-	gp59[N4]	3e-180	
**83**	**81**	68	15.1	+	dUTPase [Pelobacter]	2e-21	Deoxyuridinetriphosphatase [pfam00692, 1e-21]
**84**	**83**	44	28.0	-	gp67[N4]	1e-4	
**85**	**84**	67	64.3	-	gp68[N4]	6e-153	Terminase, large subunit
**86**	**85**	56	26.9	-	gp69[N4]	2e-41	
**-**	**88**	-	10.3	+	No similarity		Three transmembrane domains

[a] Mutual similarity between corresponding LUZ7 and LIT1 proteins is expressed as % amino acid identity. The identity of corresponding ORFs of LIT1 and PEV2 can be derived from Figure 2.

[b] Protein sequences were analysed using BLASTp against the NCBI non-redundant database.

SUPPLEMENTARY TABLE S2. Putative early promoter (P_e) and factor-independent terminator (T) sequences of the N4-like viruses infecting *P. aeruginosa*. The stem-loop structures are underlined and the conserved bases are shaded in grey.

	Phage	Strand	Start (nt)	Preceding gene	Sequence
P_{e1}	LIT1	+	117	1	TCAAGAGAAGCCTTCGGCTGTGCTGTGGAT
	PEV2	+	116	1	TCAAGAGAAGCCTTCGGCTGTGCTGTGGAT
	LUZ7	+	65	1	CCTCTGCTGGCCTTCGGCCTTGGAGCGGAT
P_{e2}	LIT1	+	3272	12	GTCCTCATGGCCTACGGCCATGGGGTGGAA
	PEV2	+	3256	12	GTACTCATGGCCTACGGCCATGGGGTGGTA
P_{e3}	LIT1	+	3531	13	GCAAGAGCAGGCTACGCCTGTGTAATGGCC
	PEV2	+	3516	13	GCAAGAGCAGGCTACGCCTGTGTAATGGCT
	LUZ7	+	3680	13	TCAAGAGCAGGCTACGCCTGTGTAATGGCA
T_1	LUZ7	+/-	4402	15	AAAACCATGTGCTAACCCATGGTTTT
T_2	LUZ7	+	6913	21	CCTCCCTTGGCTTCGGCTAAGGGAGGTTCCTTTT
T_3	LUZ7	+	8272	22	CTTCCCCTGAAAAGGGGAAGGCATCCTTTTTTT
T_4	LUZ7	+	26294	47	GGGGGAGGCAACTCCCCCACTCTTT
T_5	LUZ7	+	26972	49	AACCCCTCTTCGGAGGGGTTTTTTATT
T_6	LUZ7	-	27812	52	ATAAAAAAACCCCCTACAGGTAGCGACATGCCTGTAGGGGGTT
T_7	LUZ7	+	39352	62	GGGCCGCTTCCAGCGGCTCTTT
T_8	LUZ7	+/-	43770	72	AAAAAGAAAGGGGAGCATTAGCTCCCCTTCTTCTTT
T_9	LUZ7	-	58263	77	AAAAAGGGGGACCGAAGTCCCCCT
T_{10}	LUZ7	-	59951	79	AAAAAAGGGGAGGTTTCCCTCCCCT
T_1	LIT1	+	3926	14	GCCCACATCCTCACTCGATCCTGAGTAGGGTGTGGGCTGTTTTTTTTTTT
T_2	LIT1	+	9218	24	GCGTACTCCGTACGCTTTTT
T_3	LIT1	-	21618	40	AAACCAACAGTGCCCAAGGGCTACATCTTGGGCAC
T_4	LIT1	+/-	26575	44	AAGTAATAAACCAGGGGGAGGCAACTCCCCCTCTTTCTTT
T_5	LIT1	+	27253	45	CCCGCTTCGGCGGGTTTTTTATT
T_6	LIT1	-	28037	47	AAAAAACCCCCGAAGACCGCAGTCTAAGGGGG
T_7	LIT1	+	39591	60	GGGTGCCTCCGGCACCCTTT
T_8	LIT1	+	44507	69	GGGGGAGCAATAGCTCCCCCTTTCTT
T_9	LIT1	-	58982	73	AATAAAAAAGGGGAACCGAAGTTCCCCTT
T_{10}	LIT1	-	66397	80	AAAAAAAGAGGGGACCGTCCTGGTCCCCTC
